# Transcription factor 19‐mediated epigenetic regulation of FOXM1/AURKB axis contributes to proliferation in clear cell renal carcinoma cells

**DOI:** 10.1002/mco2.442

**Published:** 2023-12-03

**Authors:** Yakun Luo, Qing Shi, Lu Wang, Shuijie Li, Wanhai Xu

**Affiliations:** ^1^ NHC Key Laboratory of Molecular Probes and Targeted Diagnosis and Therapy Harbin Medical University Harbin China; ^2^ State Key Laboratory of Frigid Zone Cardiovascular Diseases, Department of Biopharmaceutical Sciences College of Pharmacy, Harbin Medical University Harbin China

Dear Editor,

1

Clear cell renal cell carcinoma (ccRCC) patients with metastasis and recurrence not only lose the chance of surgery, but also tolerate radiotherapy and chemotherapy.^1^ Although targeted drugs have been commonly used to treat ccRCC, resistance to the drug results in unsatisfactory long‐term effects.^1^ Therefore, exploring the molecular mechanisms underlying the development of ccRCC and mining novel targeted biomarkers are of utmost significance for guiding patient therapy. Recently, transcription factor 19 (TCF19) has exhibited the potential prognostic value and can predict the survival rate of the ccRCC patients.^2^ However, the function of TCF19 has not yet been systematically studied in ccRCC. Herein, we aimed to dissect the underlying mechanism involved in the proliferation of ccRCC cells.

We first analyzed the *TCF19* transcription expression in ccRCC tissues based on the The Cancer Genome Atlas (TCGA) database and found that the *TCF19* mRNA was significantly higher in ccRCC tissues (*n* = 532) compared to adjacent normal tissues (*n* = 100) (Figure 1A). Meanwhile, this finding was demonstrated by quantitative reverse transcription PCR (RT‐qPCR) using 29 cases of normal tissues and 45 cases of ccRCC tissues. Consistent with the above data mining analysis and previous study,^2^ significantly increased expression of *TCF19* mRNA was observed in ccRCC tissues compared to the normal tissues (Figure S1A). Furthermore, western blotting and Immunohistochemistry (IHC) staining were performed with nine paired ccRCC tissues and adjacent normal tissues, respectively. We found that the expression of TCF19 protein was significantly increased in ccRCC tissues compared to adjacent tissues (Figures 1B and S1B,C). In particular, ccRCC patients with low *TCF19* mRNA levels had longer overall survival as compared to those with high *TCF19* mRNA expression (Figure 1C), which was based on the UALCAN online database. Furthermore, Kyoto Encyclopedia of Genes and Genomes (KEGG) pathway analysis (Figure S1D), which based on the TCGA database, revealed that the upregulation pathway of TCF19 was mainly associated with cell cycle biological processes (Figure S2A,B). Moreover, compared with HK2 cells, the TCF19 was upregulated expression in ccRCC cells (786‐O, Caki‐1, and 769‐P) (Figure S1E,F). Meanwhile, it was shown that TCF19 was localized primarily in the nucleus in ccRCC cells (Figure S1G). Intriguingly, we observed that compared to the control (siCtrl), TCF19 knockdown (TCF19‐KD) via RNA interference (siTCF19) (Figure S3A,B) significantly reduced cell proliferation (Figures 1D and S3C–F) and induced cell cycle arrest (Figure S3G) in ccRCC cells.

**FIGURE 1:**
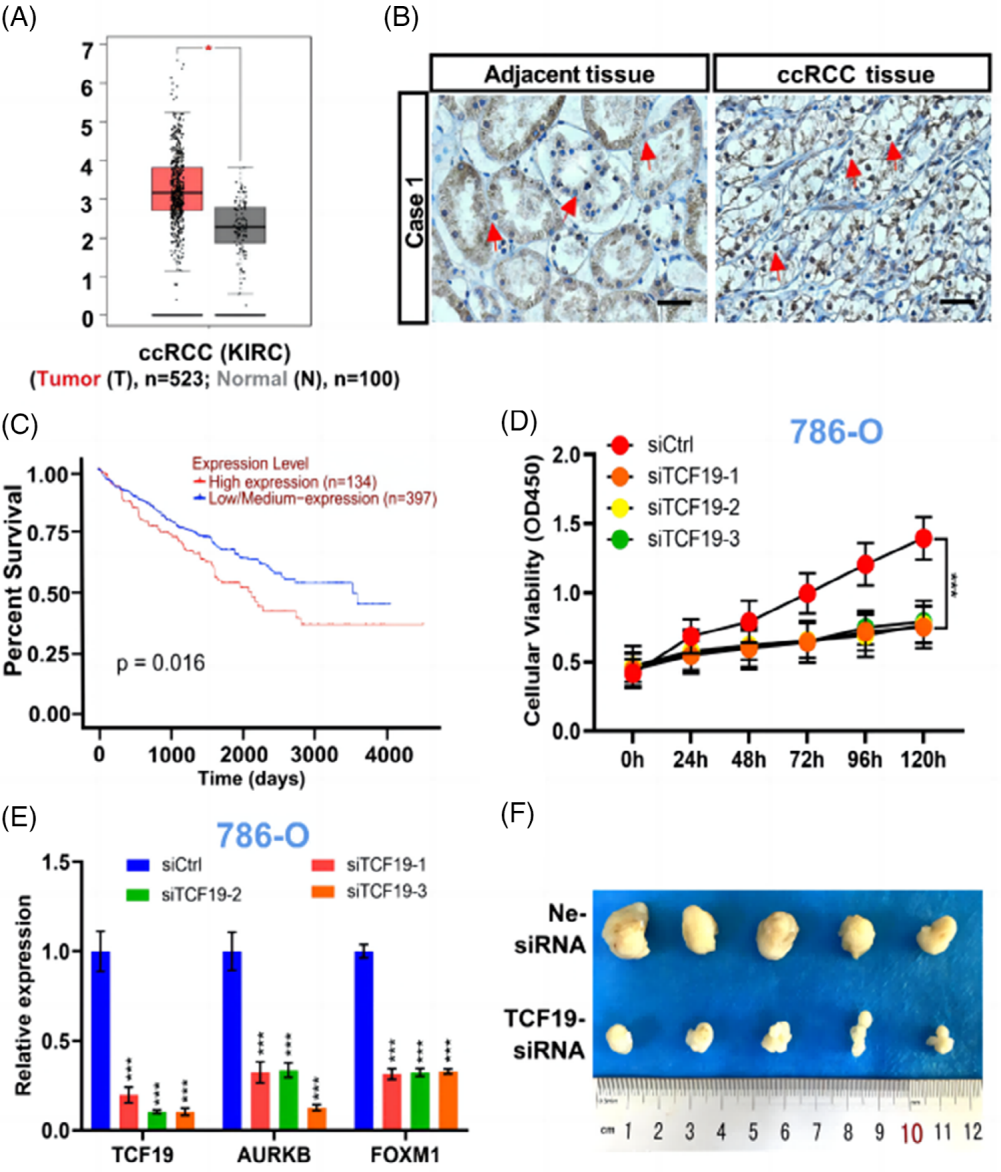
Transcription factor 19 (TCF19) promotes the cell proliferation via activating the transcription of *AURKB* and is essential for *AURKB* transcription mediated by Forkhead box protein M1 (FOXM1) in clear cell renal cell carcinoma (ccRCC) cells. (A) Based on TCGA online database, data mining showed TCF19 mRNA expression in ccRCC tissues and adjacent tissues. (B) Representative IHC images of TCF19 expression in human clinical ccRCC tumor tissues and adjacent tissues. Scale bar = 100 μm. (C) Based on the UALCAN (https://ualcan.path.uab.edu/) online database, the overall survival (OS) of ccRCC patients was analyzed. (D) KEGG analysis showing that TCF19 participates in biological processes in ccRCC. (D) Cell Counting Kit‐8 (CCK‐8) analysis showing the effect of TCF19 knockdown (TCF19‐KD) on the cell proliferation of the tested ccRCC cell lines. (E) *AURKB* and *FOXM1* mRNA expression was analyzed by RT‐qPCR in TCF19‐KD 786‐O cells. (F) Image of xenograft tumors indicating 786‐O cells in nude mice upon TCF19‐KD or negative control treatment. For all RT‐qPCR and Chromatin immunoprecipitation‐Quantitative Polymerase Chain Reaction (ChIP‐qPCR) analyses, the mean ± standard deviation (SD) (*n* = 3) is shown. Student's *t*‐test, ns: non‐significant, ^***^ *p* < 0.001 versus control. AURKB, aurora kinase B.

Next, we sought to dissect the mechanism of TCF19 regulation in the cell proliferation of ccRCC cells. Aurora kinase B (AURKB), a cell cycle regulator,^3^ is a potential therapeutic target for ccRCC.^4^ Intriguingly, the GSEA enrichment analysis (Figure S4A), protein‐protein interaction (PPI) predication (Figure S4B) and molecular docking predication (Figure S4C–E) identified that TCF19 strongly interacted with AURKB in ccRCC. Then, co‐inmunoprecipitation (co‐IP) analysis demonstrated the physical interaction between TCF19 and AURKB in ccRCC cells (Figure S4F). More intriguingly, TCF19‐KD significantly reduced the AURKB expressions at the transcriptional and protein levels (Figures 1E and S5A,B and Table S1), and we found that AURKB‐KD triggered reduced cell proliferation (Figure S6A,B) and caused the cell cycle arrest in these cells (Figure S6C–E). Several distal regulator sequences in the *AURKB* promoter have been identified,^5^ named AURKB Area 1, Area 2, Area 3, and Area 4 for this study (Figure S7A and Table S2). Importantly, ChIP‐qPCR analyses demonstrated that TCF19 bound to these four areas on the *AURKB* promoter, with the strongest binding affinity around Area 1 in these cancer cells (Figure S7B). Moreover, TCF19 was demonstrated as an H3K4me3 recognizer^6^ and our data also suggested that TCF19 exerts its downstream functions in triggering the proliferation of ccRCC cells through regulating the H3K4me3 mark enriched at the *AURKB* promoter (Figure S7C). More interestingly, ChIP‐qPCR analysis results demonstrated that the menin, a co‐activates transcription factor mediated by regulating H3K4me3,^7^ also participated in the TCF19‐regulated *AURKB* transcription in ccRCC cells (Figure S7C). Notably, *MEN1* silencing resulted in significantly decreased expression of AURKB (Figure S8A and Table S3), suppressed cell proliferation (Figure S8B), and induced cell cycle arrest at G0/G1 phase in ccRCC cells (Figure S8C). To our knowledge for the first time, in ccRCC cells, our data revealed that TCF19 is a crucial factor for positively regulating the *AURKB* transcription mediated by menin/H3K4me3 epigenetic alteration.

In parallel, we found that FOXM1, a situated upstream regulator of the AURKB,^8^ executes oncogenic activity in ccRCC cells.^9^ *FOXM1* silencing caused a strong reduction in AURKB expression in 786‐O, Caki‐1, and 769‐P cells (Figure S9A,B), similar to that observed in U2OS cells.^8^ Furthermore, we also found that FOXM1 regulates *AURKB* transcription via binding to the proximal *AURKB* promoter in ccRCC cells (Figure S9C,D). Hence, we assessed whether TCF19 is involved in the regulation of AURKB by FOXM1 in ccRCC cells. Subsequently, we first demonstrated that TCF19‐KD significantly reduced the FOXM1 expression (Figures 1E and S10A,B), and ChIP‐qPCR analysis revealed that TCF19 regulates *FOXM1* transcription, most likely via binding to the *FOXM1* promoter (Figure S10C,D), mediated by H3K4me3 histone mark (Figure S10E). Furthermore, we deeply analyzed the sequences of the *AURKB* promoter and found that a TCF19 and FOXM1 co‐binding area (named as CoBs) exists at the *AURKB* promoter. Subsequently, ChIP‐qPCR analysis demonstrated that TCF19 and FOXM1 co‐bound at the CoB area of the *AURKB* promoter in ccRCC cells (Figure S11A,B). Remarkably, FOXM1 binding to the CoBs was significantly reduced upon TCF19‐KD treatment (Figure S11C), whereas FOXM1‐KD did not affect the affinity of TCF19 binding at the CoB locus in ccRCC cells (Figure S11D). Taken together, our data demonstrated that TCF19 is essential for the FOXM1‐mediated *AURKB* transcription in ccRCC cells.

Finally, we investigated whether TCF19 has an effect on the tumorigenic capacity of ccRCC cells in vivo, 786‐O cells were subcutaneously injected into BALB/c nude mice and treated with control siRNA (Ne‐siRNA), and TCF19‐siRNA in vivo siRNA delivery as indicated (Figure S12A). In line with the observation that inhibition of 786‐O cell growth in vitro by silencing of the *TCF19* gene, the remarkable reduction in TCF19 expression (Figure S12B) and a significant suppression of tumor growth rate as well as smaller tumor volume were observed in TCF19‐siRNA 786‐O tumor xenografts compared with Ne‐siRNA group (Figures 1F and S12C). In addition, IHC staining analysis revealed lower expression of Ki67, TCF19, AURKB, and FOXM1 in TCF19‐siRNA tumors compared to the Ne‐siRNA tumors (Figure S12D). Furthermore, data mining revealed significantly positive correlations between *TCF19* and *AURKB* mRNA expression, between *TCF19* and *FOXM1* mRNA expression and between *FOXM1* and *AURKB* mRNA expression in ccRCC (Figure S13).

In summary, we unveiled that the TCF19 is a new critical factor in promoting the proliferation of ccRCC cells, involving a TCF19‐mediated epigenetic mechanism that triggers FOXM1/AURKB signaling. Our findings provide novel insights into the potential for developing a novel therapy targeting the TCF19/FOXM1/AURKB axis to treat ccRCC.

## AUTHOR CONTRIBUTIONS

Y.K.L. and Q.S. contributed to evaluating the data, preparing the figures, and reviewing the literature. L.W. provided technical and material support and participated in data interpretation. S.J.L. participated in study design and provided critical revision of the manuscript. Y.K.L. and W.H.X. conceived and supervised the study and manuscript preparation. Y.K.L. obtained funding. All the authors have read and approved the final manuscript.

## CONFLICT OF INTEREST STATEMENT

The authors declare they have no conflicts of interest.

## ETHICS STATEMENT

The study was conducted in accordance with the guidelines in the Declaration of Helsinki and the use of all specimens of patients with bladder cancer was approved by the Ethics Committee of Harbin Medical University (2022‐SCILLSC‐30) and carried out according to the application guidelines and regulations. The Harbin Medical University Animal Care and Use Committee released the approval (no. SYXK2022‐014) for the animal experiment. Written informed consent was obtained from all participants.

## Supporting information

Supporting information

## Data Availability

All data generated or analyzed during this study are included in this article.
