# Supplementary material for: Transcription factor 19‐mediated epigenetic regulation of FOXM1/AURKB axis contributes to proliferation in clear cell renal carcinoma cells
Source: MedComm (2020). 2023 Dec 3;4(6):e442. doi: 10.1002/mco2.442 (PMC10694380; doi:10.1002/mco2.442)
Supplement: Supplementary file 1 — Supporting information [file MCO2-4-e442-s001.pdf]

Supplementary Files for

Transcription Factor 19-mediated epigenetic regulation of FOXM1/AURKB axis contributes to proliferation in clear cell renal carcinoma cells

Yakun Luo [1, # *], Qing Shi [1, #], Lu Wang [1], Shuijie Li [2 *], Wanhai Xu [1 *]

1. NHC Key Laboratory of Molecular Probes and Targeted Diagnosis and Therapy, Harbin Medical University, Harbin, 150001, China;
2. State Key Laboratory of Frigid Zone Cardiovascular Diseases (SKLFZCD), Department of Biopharmaceutical Sciences, College of Pharmacy, Harbin Medical University, Harbin, 150081, China.

[#] Equal contribution: Yakun Luo and Qing Shi

* Correspondence to: Yakun Luo, Shuijie Li and Wanhai Xu

Running Title: ***TCF19 promotes ccRCC progression via targeting FOXM1/AURKB axis***

Supplementary Materials and Methods

1. Clinical samples

A total of 45 of clear cell renal carcinoma (ccRCC) tissues or 29 of adjacent tissues were collected from the Department of Pathology, The 4th Affiliated Hospital of Harbin Medical University (Harbin, China), from December 2015 to Mar 2021. All patients were first diagnosed with ccRCC and then underwent radical nephrectomy with curative intent. No preoperative chemotherapy or radiotherapy was performed. The 9 pairs of ccRCC and para-carcinoma tissues were selected for IHC detection, western blotting or RT-qPCR analysis. This research was carried out in accordance with the World Medical Association Declaration of Helsinki and was approved by the Harbin Medical University Research Ethics Committee.

2. Raw data for mining analysis

The GEPIA (Gene Expression Profiling Interactive Analysis) for cancer genomics (http://http://gepia.cancer-pku.cn/), a recognized and widely used online portal providing analysis of large-scale cancer genomics data sets [1], was applied to analyze mRNA expression of *TCF19* in ccRCC samples. Meanwhile, we used the LinkedOmics (http://www.linkedomics.org) online software, which was applied to analyze mRNA co-expression between *TCF19* and *AURKB*, *TCF19* and *FOXM1*, *TCF19* and *CDK1*, *TCF19* and *CDK2, TCF19* and *CCNA2, TCF19* and *CCNB1, TCF19* and *CCNE1, TCF19* and *MYC, AURKB* and *FOXM1* in ccRCC. Meanwhile, we also used the LinkedOmics online database [2] to analyze functional and pathway enrichment (KEGG pathway maps) of TCF19 in ccRCC. Furthermore, we also applied UALCAN (https://ualcan.path.uab.edu/) online software to predict the correlation between *TCF19* mRNA expression and overall survival (OS) of ccRCC (or KIRC).

3. Cell culture

786-O, Caki-1 and 769-P cells were purchased form American Type Culture Collection (ATCC) (Manassas, VA, USA). Cells were cultured with RPMI 1640 medium (11875176, Gibco), which was supplemented with 10% fetal bovine serum (FBS)(10099141C , Gibco) and 1% antibiotic (mixtures of penicillin and streptomycin) (10378016, Gibco). Cells cultured was carried out at 37 ℃ in a 5% CO_2 humidified environment.

4. Cell transfection

Before the experiment, 786-O, Caki-1 or 769-P cells were seeded into 6-well plates with an appropriate density, respectively. Small interfering RNAs (siRNA), including siTCF19-1 (#stB0005746A-1-5), siTCF19-2 (#stB0005746B-1-5), siTCF19-3 (#stB0005746C-1-5), siFOXM1-1 (#stB0001139A-1-5), siFOXM1-2 (#stB0001139B-1-5), siFOXM1-3 (#stB0001139C-1-5), siAURKB-1 (#stB0005033A-1-5), siAURKB-2 (#stB0005033B-1-5) and siAURKB-3 (#stB0005033C-1-5) were purchased from Ribo Bio (Guangzhou, China), which were then transiently transfected in 786-O, Caki-1 or 769-P cells using transfection reagent Interferrin (114-15, Polyplus) following the manufacture's instructions. Non-targeting control pool (siCtrl) from Sigma-MERCK was used as a negative control for

each RNA interference experiment. After 72 h post-transfection, western blot was used to detected the efficiency of gene knockdown. Subsequently, further experiments were carried out, including assays for cell proliferation, colony formation, cell flow cytometry, qRT-PCR and western blotting.

5. CCK-8 assay

Cell proliferation was determined with CCK-8 assay. Briefly, cells were plated in 96-well plates at a density of 2× 10^3 cells per well and incubated in a 37℃, 5% CO_2 incubator. Each sample was ste with 4 replicates wells. After cultured for 12, 24, 48, 72 and 96 h, we added 8 μL CCK-8 solution (GK10001, GlpBio) and 92 μL serum free medium to each well, and then detected after incubated for 3 h. Light absorbance at 450 nm was measured daily with an enzyme-linked analyzer, FLx8 (BioTek, Vermont, USA).

6. Colony fromation

Cells were seeded in a density of 500 cells per well into 6-well plate and cultured in for 2 weeks at 37℃ in a 5% CO_2 humidified environment. Colonies were then fixed with methanol, stained with crystal violet (0.5% w/v), and counted. Each experiment with duplicates was performed independently at least.

7. Cell cycle analysis

For cell cycle analysis, cell flow cytometry was used to examine ccRCC cells. Briefly, cells were collected and fixed in 70% ethanol at 4 ℃ overnight followed by staining with propidium iodide (PI), and were analyzed using FACs flow cytometer (Becton-Dickinson, Mountain View, CA). For each sample, 20,000 cells were analyzed with CellQuest software. Cell cycle distribution was analyzed and cells in G1, S or G2/M-phase were counted both by ModFit software.

8. RNA exaction and Real time PCR analysis

Trizol reagent was used to isolate the total RNA of ccRCC tumor samples, and RNA was converted into cDNA with a special cDNA synthesis kit according to the manufacture's protocol. Human gene expression was measured using RT-QPCR on the ABI 7900 system. Expression of target genes was normalized with the expression of beta-actin. The primer sequences were listed in **Supplementary Table S1**.

9. Flow cytometry analysis

Cells were treated with siCtrl or RNA interfering (siRNA) as experiment indicated for 72 h, followed by harvesting and re-suspending in 100 μL of phosphate-buffered saline (PBS, Gibco) and fixed with 70% ethanol at 4°C overnight, and stained with propidium iodide (50 μg/mL) containing 100 μg/mL RNase A for 15 min. Finally, the cell cycle analyses were performed via flow cytometry on a FACScan instrument (Beckman Coulter, Brea, California, USA).

10. Chromatin immunoprecipitation (ChIP)-qPCR analysis

The detail of ChIP-qPCR procedure was mentioned previously [3]. The Millipore ChIP Assay Kit (Catalog. 17-295, Millipore, Massachusetts, USA) was employed to performed the ChIP. Each ChIP assay was repeated at least three times independently. Primers used for ChIP-qPCR are listed in **Supplementary Table S2**.

11. Protein extraction and western blot analysis

Toatal protein from cells or frozen tissues samples was extracted from cells using RIPA lysis buffer (Catalog. 89900, Thermo Fisher) and quantified with a Protein BCA Assay kit (Bio-RAD).The protein was then separated by sodium dodecyl sulfatepolyacrylamide gel eletrophoresis (SDS-PAGE) and transferred to a polyvinylidence difluoride (PVDF) membrane (Millpore Corporation, USA).The membrane was then blocked with 5% powdered milk at room temperature for 1 h, followed by incubation overnight at 4 ℃ with mouse anti-TCF19 and anti-β-actin antibodies as indicated. After incubated with horseradish peroxidase-conjugated secondary antibodies, target protein bands were visualized using the enhance chemiluminescence method in a ChemiScope3400 imaging system. Primer antibodies uesd were listed in **Supplementary Table S3**.

12. PPI network construction and docking analysis

We used Search Tool for the Retrieval of Interacting Genes (STRING) Database (STRING) (http://www.string-db.org/) to assess protein-protein interaction (PPI) information.

For the Protein-protein docking analysis, the AlphaFold-predicted structures of TCF19 and AURKB were downloaded from the Uniprot database (https://www.uniprot.org/) with access IDs Q9Y242 and Q96GD4, respectively. Low-confident regions in these predicted structures were refined via molecular dynamics (MD) simulations using the Amber20 package. The protein was described by the Amber FF19SB force field and was energy-minimized. The system was then heated from 0 to 300 K using Langevin dynamics in an implicit solvent environment. The MD refinement simulation lasted 400 ns, and the last frame was selected as input structures for protein-protein docking using the ZDOCK online server (https://zdock.umassmed.edu/). The top ten docked TCF19-AURKB complexes were subject to 20 ns MD refinement following the above-mentioned protocol. The MMPBSA.py script from Amber20 package was used to calculate the binding free energies.

13. Co-immunoprecipitation

Co-immunoprecipitation (Co-IP) assays were performed as described before [3]. Co-IP reaction was made overnight with 1 mg of cell extract, dissolved in 500 μL of lysis buffer (Catalog. 89900, Thermo Fisher) supplemented with protease inhibitors (Catalog. 11836145001, Roche Molecular Biochemicals, Basel, Switzerland). Cellular lysates were incubated with either anti-TCF19 or rabbit IgG antibodies overnight at 4°C, and further with protein A-agarose (Catalog. 05015979001, Roche) for 2 h.

14. Immunofluorescence (IF) staining

For IF staining, cells were grown on glass coverslips, then fixed with methanol for 5 min at room temperature. Following fixation, the cells were blocked with Dako buffer (Catalog. S0809, Agilent, California, United States) for 1 h, and incubated with primary antibodies overnight at 4°C, then with appropriate secondary antibodies conjugated with Alexa 555 (red) or Alexa 488 (green) (Cell Signaling Tech). Cells were counterstained with DAPI (Catalog. #DUO82040, Sigma-Aldrich) for 10 min and visualized by fluorescence microscopy. The following primary antibodies were used for immunostaining: rabbit anti-TCF19 (1/1000) (Catalog. sc-390923, Santa Cruz Biotech).

15. Immunohistochemistry (IHC) analysis

Formalin fixed paraffin embedded slides were deparaffinized in Xylene, 100% ethanol and 95% ethanol. Antigens were retrieved by boiling slides in 10mM sodium citrate for 10 min. After cooling, slides were treated with 3% H_2O_2 for 30 min followed by PBS + 1% Tween 20 washing and blocked with serum. Slides were then incubated with 1:500 TCF19 antibody for 2 hours at room temperature, washed three times with PBS buffer, incubated with biotinylated goat anti-rabbit IgG secondary antibody for 1 hour at room temperature. After washing with biotin-avidin solution for 30 min at room temperature. Slides were rinsed with PBS three times, DAB solution was added to allow color development for 2 min.

16. Xenograft model and treatment

All animal experiments were performed in accordance with protocols approved by the Animal Care and Use Committee of Harbin Medical University. Female BALB/c nude mice (6-weeks-old; body weight 20-25g; n=10) were obtained from Vital River Laboratories (Beijing, China) and maintained at 20-26 °C and 40–70% humidity with specific pathogen-free conditions. The mice were divided into control (Ne-siRNA, n=5) and the *TC19* gene inactivation (TCF19-siRNA, n=5) groups. Each nude mouse was injected with 100 μL (1.0×10^7 cells) of 786-O cells. The formula length × width ^{2} × 0.5 was used to calculate the tumor volume. 40 days after inoculation, the xenografts were excised, and their volume was determined. The *in vivo* RNA interfering delivery of knockdown *TCF19* (TCF19-siRNA) or control (Ne-siRNA) were purchased from Ribo Bio Technology (Guangzhou, China).

17. Statistical analysis

Each biological experiment was independent repeated at least three times to ensure accuracy, with each containing at least three technical replicates for both RT-qPCR, ChIP-qPCR and cell proliferation assays. Each variable was analyzed with the unpaired Student’s t-test compare two groups of independent samples, using the Prism 5 software (GraphPad Software). Results are expressed as mean S.E.M. P-values of < 0.05 were considered to be significant. Statistical significance is displayed as ns, non-significant vs control, *P<0.05, **P<0.01, ***P<0.0001 for all analyses.

Supplementary Tables

Supplementary Table S1. Primers used for RT-qPCR

TCF19	F: 5′ -GTCCGACTCCCAAGAGGTCA-3′<br>R: 5′ -GCAGCAAAGTCCTGAGGCT-3′	**[4]**
AURKB	F: 5′ -ATCAGCTGCGCAGAGAGATCGAAA-3′<br>R: 5′ -CTGCTCGTCAAATGTGCAGCTCTT-3′	**[5]**
FOXM1	F: 5′ -CACCCCAGTGCCAACCGCTACTTG-3′<br>R: 5′ -AAAGAGGAGCTATCCCCTCCTCAG-3′	**[6]**
HPRT	F: 5′ -TATGGCGACCCGCAGCCCT-3′<br>R: 5′ -CATCTCGAGCAAGACGTTCAG-3′	**[3]**
CDK1	F: 5′ -CCATACCCATTGACTAACTAT-3′<br>R: 5′ -ACCCCTTCCTCTTCACTTTC-3′	**[7]**
CCNA	F: 5′ -CGCTGGCGGTACTGAAGTC-3′<br>R: 5′ -GAGGAACGGTGACATGCTCAT-3′	**[8]**
CCNB1	F: 5′ -TGCCCCTGCAGAAGAAGACCTGTGT-3′<br>R: 5′ -TGTTTCCAGTGACTTCCCGACCCAGT-3′	**[9]**
CCNE	F: 5′ -GACCGGTATATGGCGACACAAGAA-3′<br>R: 5′ -GTCGCACCACTGATACCCTGAAAC-3′	**[10]**

Supplementary Table S2. Primers used for ChIP-qPCR

Primers'name	Sequence
AURKB promoter Area 1	F: 5′ -CCCTTCTCATTCCGCCTCTTC-3′
	R: 5′ -CAAACAACTGAATCGCCACGC-3′
AURKB promoter Area 2	F: 5′ -GGCCGGGCACGGTGGCTCACG-3′
	R: 5′ -CGGCTTTTCGCTGTGTTGGCC-3′
AURKB promoter Area 3	F: 5′ -GTGGGCCCAAACAACACGGCAG-3′
	R: 5′ -CTTGTGTCTATTTCTAGAC-3′
AURKB promoter Area 4	F: 5′ -GGCTCACGCCTGTAATCCAAG-3′
	R: 5′ -CAACCTCCTCCTCCAGGGTGG-3′
AURKB promoter FBs1	F: 5′ -GACGAGCTTGCCCAATGGGGC-3′
	R: 5′ -GTCCAAGGCACTGCTACTCTC-3′
AURKB promoter FBs2	F: 5′ -GGTACTCGGGAGGCTGAGACAG-3′
	R: 5′ -AGATTCGCTGTGTTGCCCAGG-3′
FOXM1 promoter BS1	F: 5′ -GCAGACCGCACAGCCTTCGAG-3′
	R: 5′ -GTGGAAGCCTGTGCGGTCTGC-3′
FOXM1 promoter BS2	F: 5′ -GCAGAGGAGCCTGAGGGGAAG-3′
	R: 5′ -GGACCGCAGGCCGGCTTGTGG-3′
FOXM1 promoter BS3	F: 5′ -GCACTACGGTCTATTATATCC-3′
	R: 5′ -CCACAGTGAACGATCGTCTGC-3′
FOXM1 promoter BS4	F: 5′ -GGCTGGAGTGCAGTGGTGTGA-3′
	R: 5′ -CTCACGCCTGTAATCCCAGCAC-3′
AURKB promoter CoBs	F: 5′ -GGGCGGGAGATTTGAAAAGTC-3′
	R: 5′ -CTCTCCCGGCCGCCCGCAAACA-3′

Supplementary Table S3. Antibodies used for this study

Name	Catalog number	Company
Mouse anti-TCF19	sc-390923	Santa Cruz BioTech
Rabbit anti-CDK1	PA5-82086	Invitrogen
Rabbit anti-CDK2	#18048	Cell signaling Tech
Rabbit anti-CDK4	#12790	Cell signaling Tech
Rabbit anti-Cyclin A2	#67955	Cell signaling Tech
Rabbit anti-Cyclin B1	#12231	Cell signaling Tech
Rabbit anti-Cyclin D1	#55506	Cell signaling Tech
Rabbit anti-Cyclin E1	#20808	Cell signaling Tech
Rabbit anti-AURKB	#3094	Cell signaling Tech
Rabbit anti-β-actin	#4970	Cell signaling Tech
Rabbit anti-FOXM1	#5436	Cell signaling Tech
Normal Rabbit IgG	#2729	Cell signaling Tech
Rabbit anti-H3K4me3	#9751	Cell signaling Tech
Rabbit anti-WDR5	#13105	Cell signaling Tech
Rabbit anti-menin	A300-105A	Bethyl Tech

References

1. Tang Z, Kang B, Li C, Chen T, Zhang Z. GEPIA2: an enhanced web server for large-scale expression profiling and interactive analysis. Nucleic Acids Res. 2019 Jul 2;47(W1): W556-W560. doi: 10.1093/nar/gkz430. PMID: 31114875; PMCID: PMC6602440.

2. Vasaikar SV, Straub P, Wang J, Zhang B. LinkedOmics: analyzing multi-omics data within and across 32 cancer types. Nucleic Acids Res. 2018 Jan 4;46(D1): D956-D963. doi: 10.1093/nar/gkx1090. PMID: 29136207; PMCID: PMC5753188.

3. Luo Y, Vlaeminck-Guillem V, Teinturier R, Abou Ziki R, Bertolino P, Le Romancer M, Zhang CX. The scaffold protein menin is essential for activating the MYC locus and MYC-mediated androgen receptor transcription in androgen receptor-dependent prostate cancer cells. Cancer Commun (Lond). 2021 Dec;41(12):1427-1430. doi: 10.1002/cac2.12217. Epub 2021 Dec 1. PMID: 34850609; PMCID: PMC8696212.

4. Xu G, Zhu Y, Liu H, Liu Y, Zhang X. LncRNA MIR194-2HG Promotes Cell Proliferation and Metastasis via Regulation of miR-1207-5p/TCF19/Wnt/β-Catenin Signaling in Liver Cancer. Onco Targets Ther. 2020 Oct 6; 13:9887-9899. doi: 10.2147/OTT.S264614. PMID: 33116574; PMCID: PMC7547811.

5. Tanaka K, Yu HA, Yang S, Han S, Selcuklu SD, Kim K, Ramani S, Ganesan YT, Moyer A, Sinha S, Xie Y, Ishizawa K, Osmanbeyoglu HU, Lyu Y, Roper N, Guha U, Rudin CM, Kris MG, Hsieh JJ, Cheng EH. Targeting Aurora B kinase prevents and overcomes resistance to EGFR inhibitors in lung cancer by enhancing BIM- and PUMA-mediated apoptosis. Cancer Cell. 2021 Sep 13;39(9):1245-1261.e6. doi: 10.1016/j.ccell.2021.07.006. Epub 2021 Aug 12. PMID: 34388376; PMCID: PMC8440494.

6. Nakamura S, Hirano I, Okinaka K, Takemura T, Yokota D, Ono T, Shigeno K, Shibata K, Fujisawa S, Ohnishi K. The FOXM1 transcriptional factor promotes the proliferation of leukemia cells through modulation of cell cycle progression in acute myeloid leukemia. Carcinogenesis. 2010 Nov;31(11):2012-21. doi: 10.1093/carcin/bgq185. Epub 2010 Sep 7. PMID: 20823107.

7. Shao Z, Li C, Wu Q, Zhang X, Dai Y, Li S, Liu X, Zheng X, Zhang J, Fan H. ZNF655 accelerates progression of pancreatic cancer by promoting the binding of E2F1 and CDK1. Oncogenesis. 2022 Aug 4;11(1):44. doi: 10.1038/s41389-022-00418-2. PMID: 35927248; PMCID: PMC9352668.

8. Guo W, Yu H, Zhang L, Chen X, Liu Y, Wang Y, Zhang Y. Effect of hyperoside on cervical cancer cells and transcriptome analysis of differentially expressed genes. Cancer Cell Int. 2019 Sep 9; 19:235. doi: 10.1186/s12935-019-0953-4. PMID: 31516392; PMCID: PMC6734331.

9. Regl G, Kasper M, Schnidar H, Eichberger T, Neill GW, Ikram MS, Quinn AG, Philpott MP, Frischauf AM, Aberger F. The zinc-finger transcription factor GLI2 antagonizes contact inhibition and differentiation of human epidermal cells. Oncogene. 2004 Feb 12;23(6):1263-74. doi: 10.1038/sj.onc.1207240. PMID: 14691458.

10. Liu C, Zhai X, Zhao B, Wang Y, Xu Z. Cyclin I-like (CCNI2) is a cyclin-dependent kinase 5 (CDK5) activator and is involved in cell cycle regulation. Sci Rep. 2017 Jan 23; 7: 40979. doi: 10.1038/srep40979. Erratum in: Sci Rep. 2017 Mar 15; 7: 44164. PMID: 28112194; PMCID: PMC5256034.

Supplementary Figures and legends

Supplementary Figure S1.

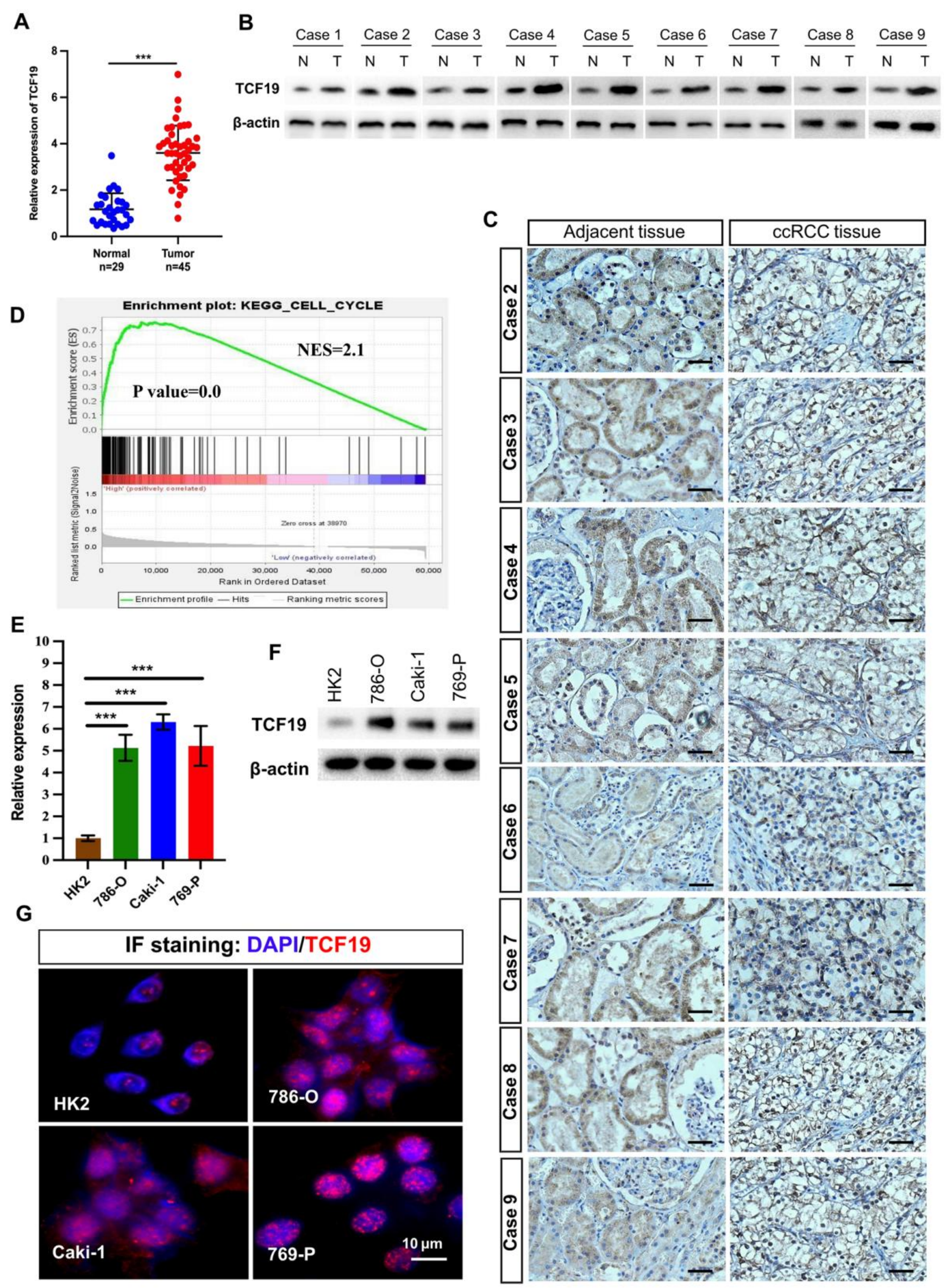

Supplementary Figure S1. High expression of TCF19 correlates with malignant progression in ccRCC.

(A) RT-qPCR analysis showing *TCF19* mRNA level in human clinical ccRCC tumor tissues (n=45) and adjacent normal tissue (n=29). **(B)** Analysis of the TCF19 protein expression in ccRCC tissues or adjacent tissues by western blotting, respectively. **(C)** IHC staining analyzed the TCF19 expression in ccRCC tissues (named as T) and adjacent tissues (named as N) which we selected form the Fourth Affiliated Hospital of Harbin Medical University. Scale bar = 100 μm. **(D)** KEGG analysis showing the TCF19 participates the biological process in ccRCC. **(E and F)** Analysis the TCF19 mRNA or protein expression in ccRCC cell lines (786-O, Caki-1 and 769-P) or human kidney cells (HK2), respectively. **(G)** IF analyzed the sub-location of TCF19 protein in 786-O, Caki-1 and 769-P or HK2 cells. ns: no significant, * $P < 0.05$, ** $P < 0.01$, *** $P < 0.001$ vs control.

Supplementary Figure S2.

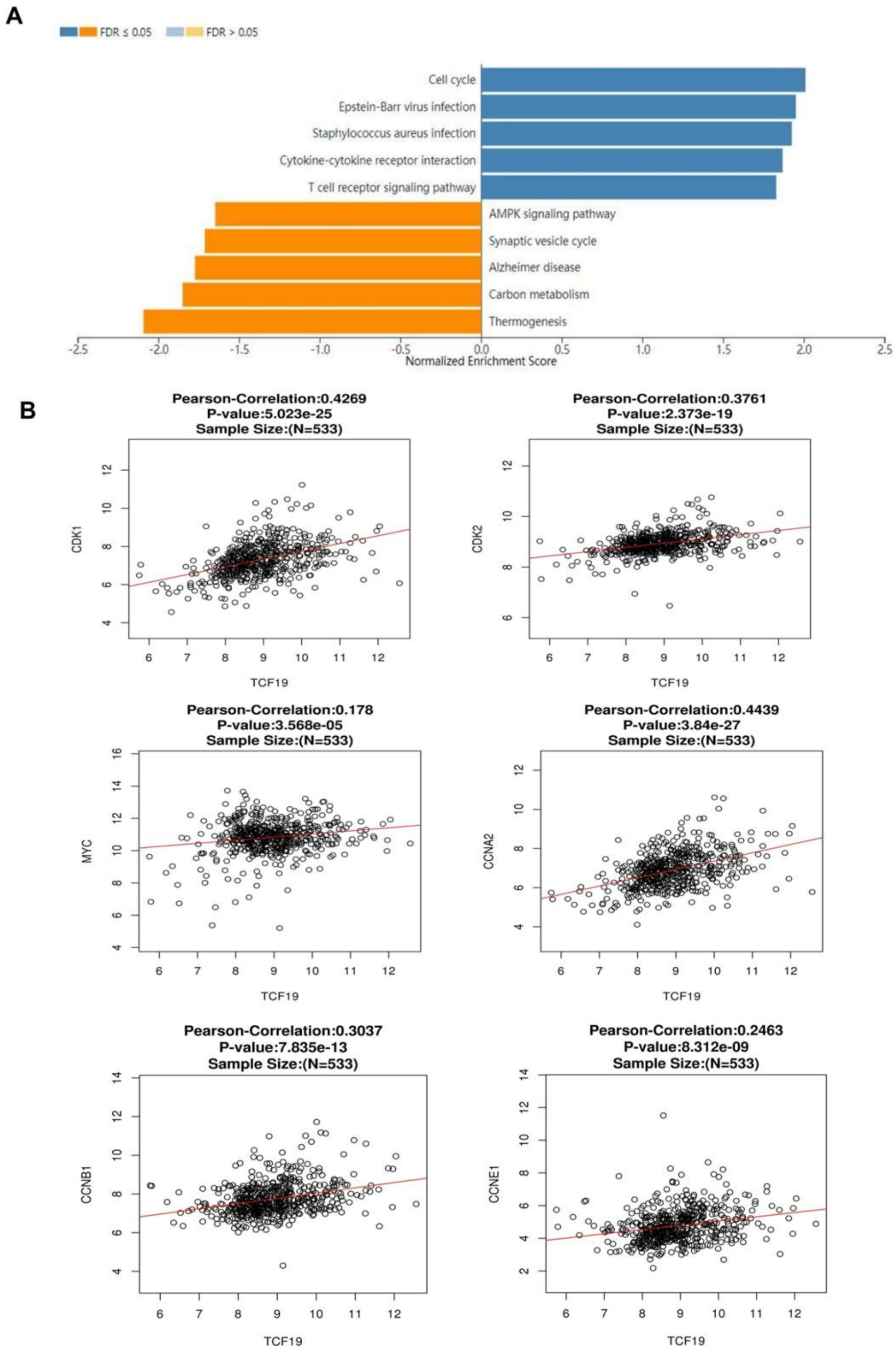

Supplementary Figure S2. TCF19 promotes ccRCC via elevating cell cycle progression.

(A) KEGG analysis showing the TCF19 participates the biological process in ccRCC which based on the Linkedomics online database (http://www.linkedomics.org). **(B)** Based on the GEPIA online database, data mining analyzed the correlation between *TCF19* mRNA expression and *CDK1, CDK2, CCNA2, CCNB1, CCNE1* or *MYC* mRNA expression in TCGA-ccRCC samples, respectively.

Supplementary Figure S3.

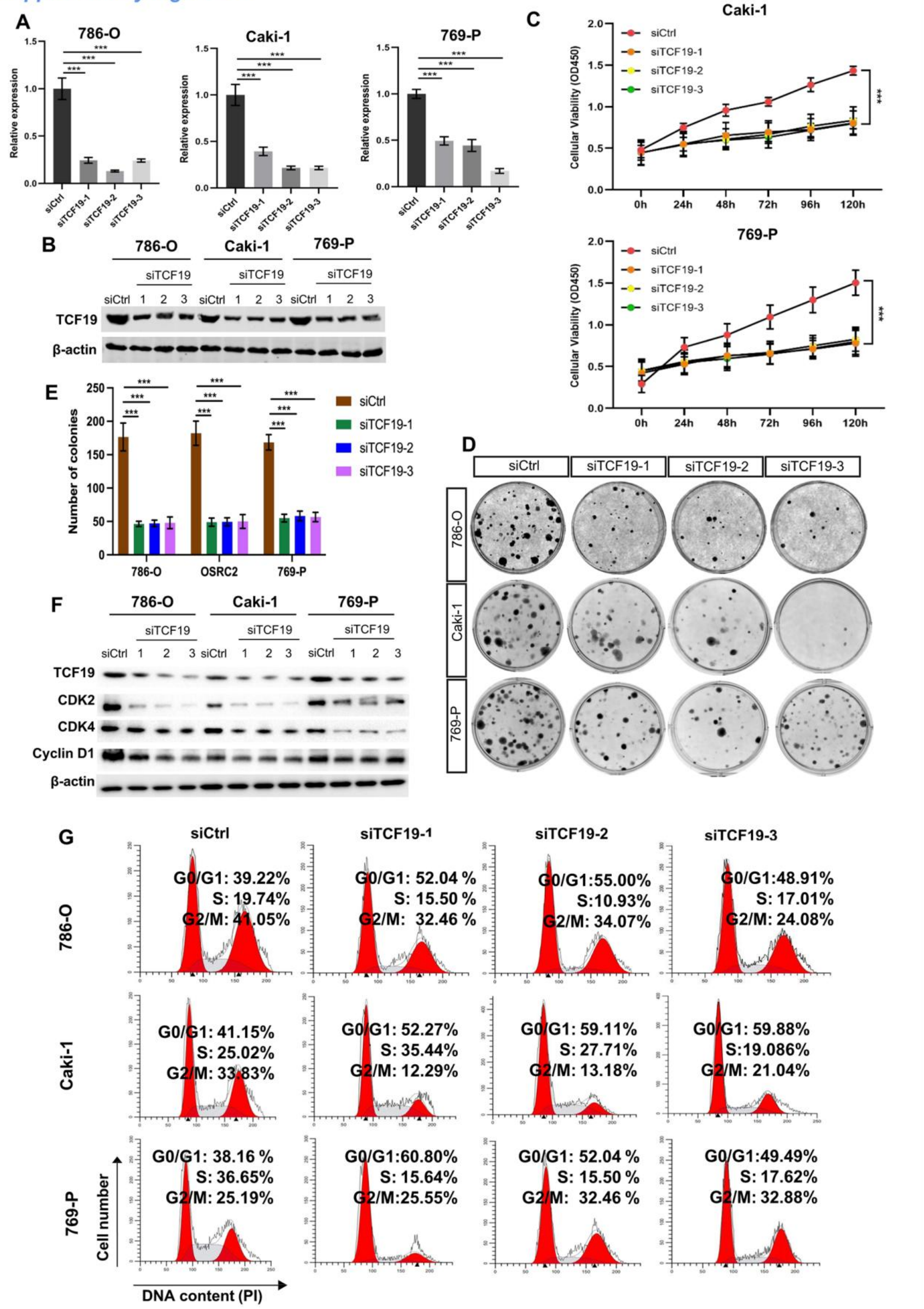

Supplementary Figure S3. TCF19 is crucial for the proliferation of ccRCC cells.

(A) RT-qPCR analyzed the efficiency of knock down the *TCF19* gene (TCF19-KD) by RNA interfering (siTCF19-1, siTCF19-2 and siTCF19-3) or control (siCtrl) in 786-O, Caki-1 and 769-P cells, respectively. **(B)** Western blotting analyzed the reduction of TCF19 protein upon TCF19-KD or siCtrl treatment in ccRCC cell lines. **(C)** CCK-8 analysis showing the effect of TCF19-KD (siTCF19-1, siTCF19-2 and siTCF19-3) on the cell proliferation of the tested ccRCC cell lines. **(D)** Representative images of colony formation assays in TCF19-KD 786-O, Caki-1 and 769-P cells. **(E)** Quantification for colonies formation in TCF19-KD or siCtrl of 786-O, Caki-1 and 769-P cells. **(F)** Western blotting showing TCF19, CDK2, CDK4 and Cyclin D1 expression in siCtrl or siTCF19-1, siTCF19-2 and siTCF19-3-treated 786-O, Caki-1 and 769-P cells. **(G)** TCF19-KD with siTCF19-1, siTCF19-2 and siTCF19-3 induces cell cycle arrest at the G0/G1 phase in 786-O, Caki-1 and 769-P cells, compared to the cells transfected with siCtrl treated cells by flow cytometry. Percentages (%) of cell populations at different stages of cell cycles are listed within the panels. The experiment was repeated three times, and representative results are presented. ns: no significant, * $P < 0.05$, ** $P < 0.01$, *** $P < 0.001$ vs control.

Supplementary Figure S4.

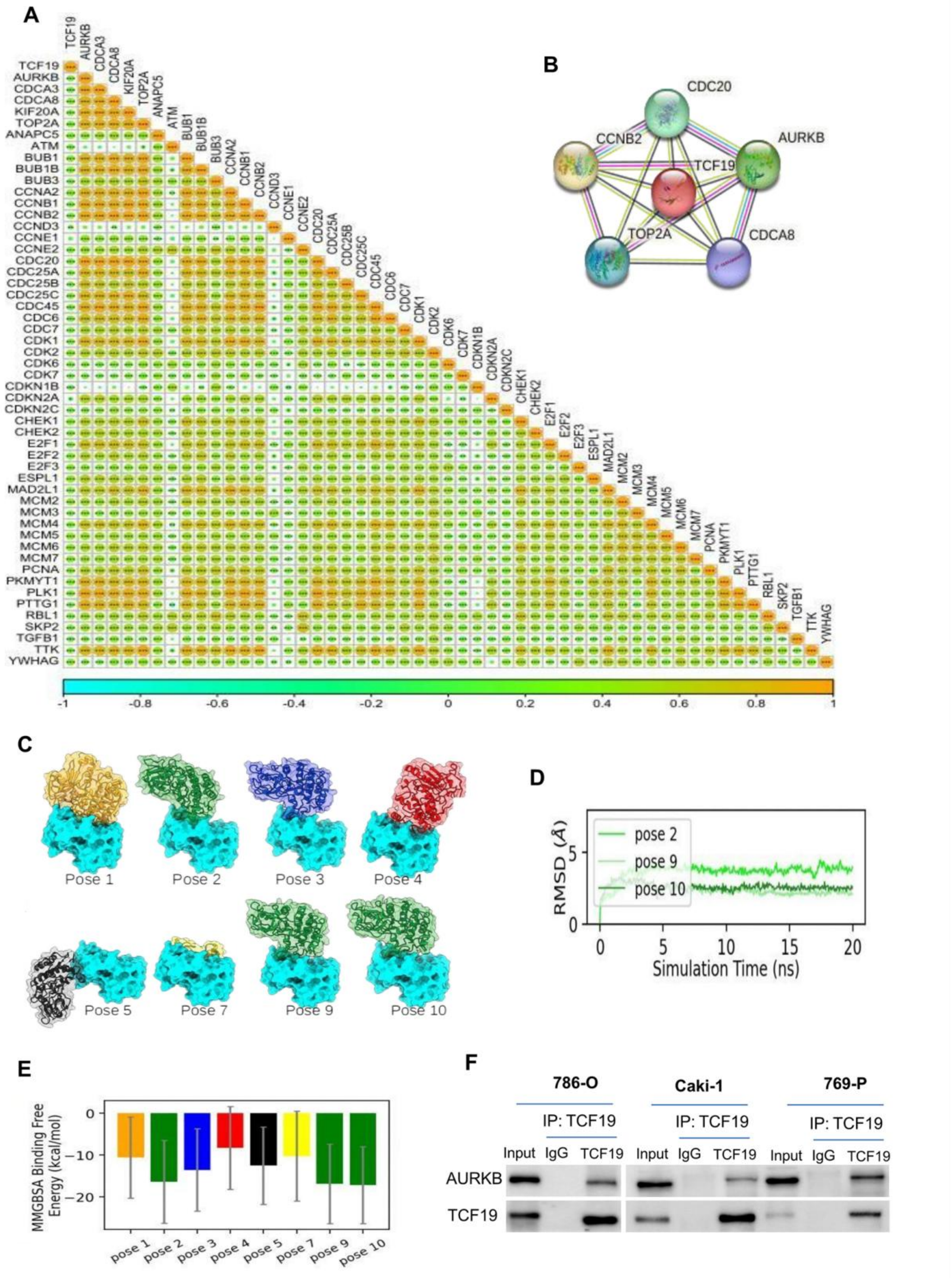

Supplementary Figure S4. AURKB is a potential target of TCF19 in ccRCC cells.

(A) GSEA enrichment analysis showing the correlation between TCF19 and cell cycle regulators, which based on the TCGA-KIRC samples. **(B)** PPI analysis for protein-protein interactions among protein targets of TCF19 through STRING online database. Each node repredents a protein and each edge represents an association. **(C** Top 10 binding poses between TCF19 and AURKB as predicted by ZDOCK online server. **(D)** Molecular dynamics simulations show that poses 2, 9 and 10 are stable as evidenced by <5 Å RMSD. **(E)** TCF19 and AURKB in poses 2, 9 and 10 have most favourable binding free energies. **(F)** Representative images of the physical interaction between TCF19 and AURKB in 786-O, Caki-1 and 769-P cells was detected by co-IP assay.

Supplementary Figure S5.

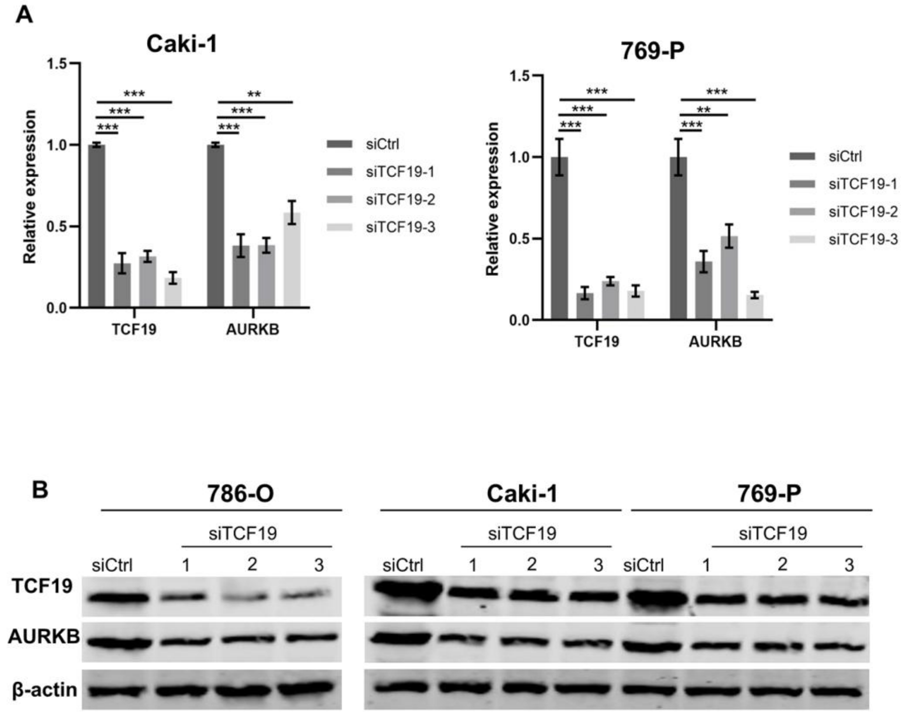

Supplementary Figure S5. TCF19 regulates AURKB expression in ccRCC cells.
(A) and **(B)** The mRNA and protein expressions of *AURKB* was analyzed by RT-qPCR and western blotting in TCF19-KD Caki-1 and 769-P cells, respectively. ns: no significant, * P < 0.05, ** P < 0.01, *** P < 0.001 vs control.

Supplementary Figure S6.

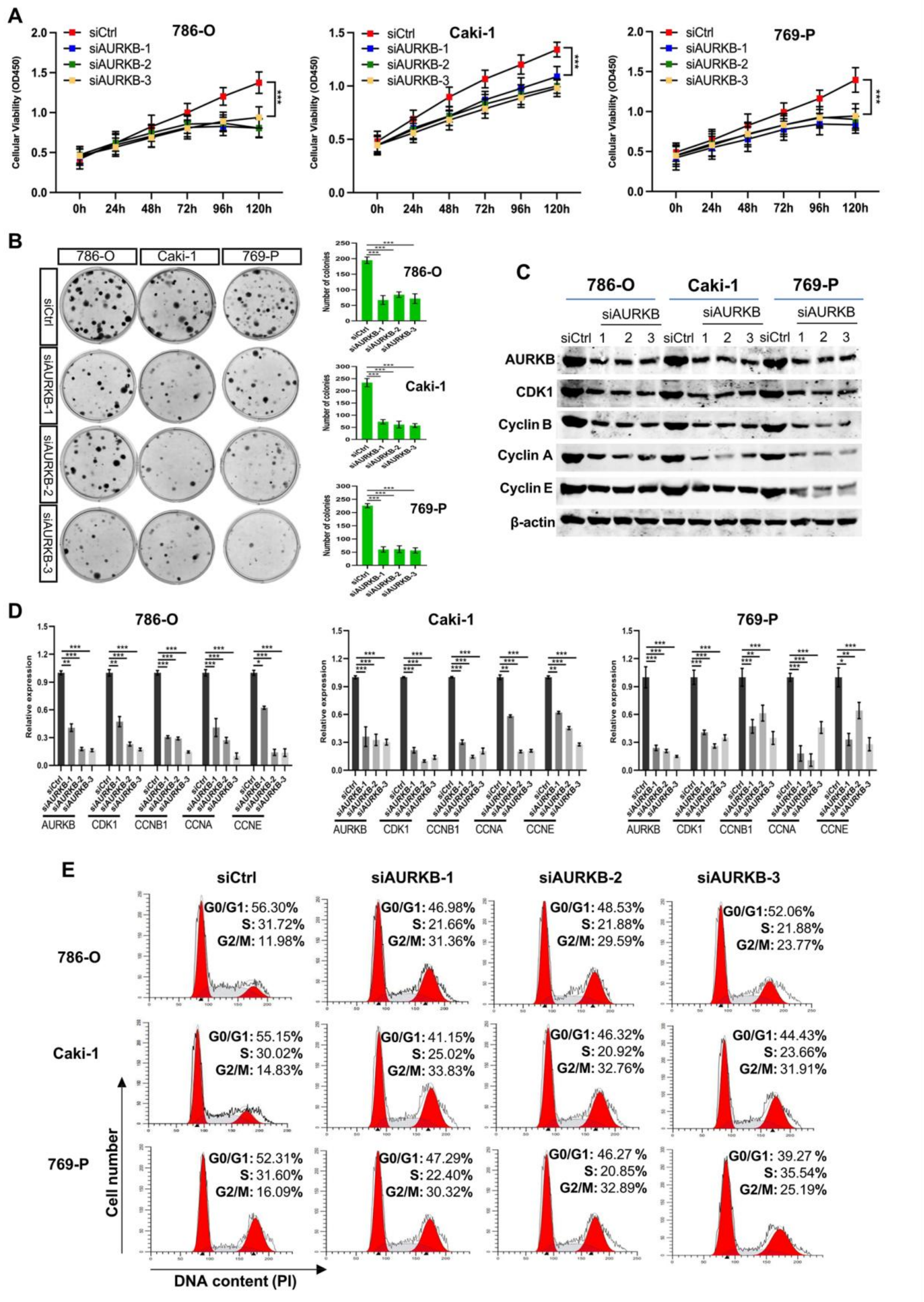

Supplementary Figure S6. AURKB-KD reduced the proliferation in ccRCC cells.

(A) CCK-8 analysis showing the effect of AURKB-KD (AURKB-1, siAURKB-2 and siAURKB-3) on the cell proliferation of the tested ccRCC cell lines. **(B)** Representative images of colonies formation assay (left panel) and their quantification (right panel) in AURKB-KD 786-O, Caki-1 and 769-P cells. **(C)** Upon AURKB-KD with siAURKB-1, siAURKB-2 and siAURKB-3-treated, RT-qPCR analysis showing the *CDK1*, *CCNB1*, *CCNA*, *CCNE* mRNA expression in ccRCC cells. **(D)** Western blotting analysis showing CDK1, Cyclin B1, Cyclin A, Cyclin E expression in AURKB-KD ccRCC cells. **(E)** AURKB-KD with siAURKB-1, siAURKB-2 and siAURKB-3 induces cell cycle arrest at the G2/M phase in 786-O, Caki-1 and 769-P cells, compared to the cells transfected with siCtrl by flow cytometry. Percentages (%) of cell populations at different stages of cell cycles are listed within the panels. The experiment was repeated three times, and representative results are presented. ns: no significant, * $P < 0.05$, ** $P < 0.01$, *** $P < 0.001$ vs control.

Supplementary Figure S7.

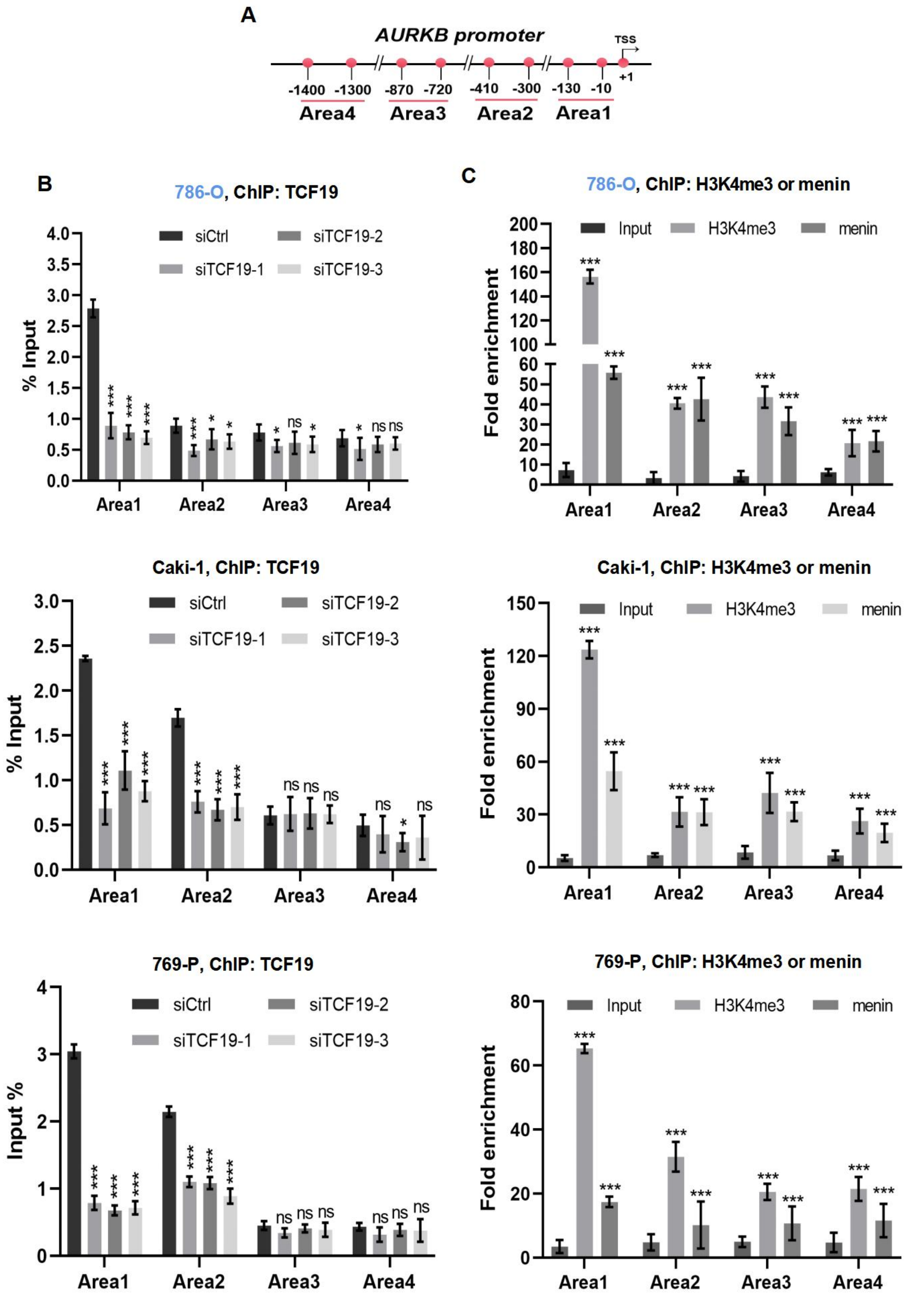

Supplementary Figure S7. A TCF19-mediated epigenetic mechanism that regulates AURKB expression in ccRCC cells.

(A) Schematic representation of the TCF19 binding site on the *AURKB* promoter. **(B)** ChIP-qPCR showing the binding of TCF19 to the *AURKB* promoter in siTCF19-1, siTCF19-2, siTCF19-3 or siCtrl-treated 786-O, Caki-1 and 769-P cells using anti-TCF19 antibody. **(C)** ChIP-qPCR evaluating the abundance of H3K4me3 histone mark or menin on the *AURKB* promoter in 786-O, Caki-1 and 769-P cells. ns: no significant, * $P < 0.05$, ** $P < 0.01$, *** $P < 0.001$ vs control.

Supplementary Figure S8.

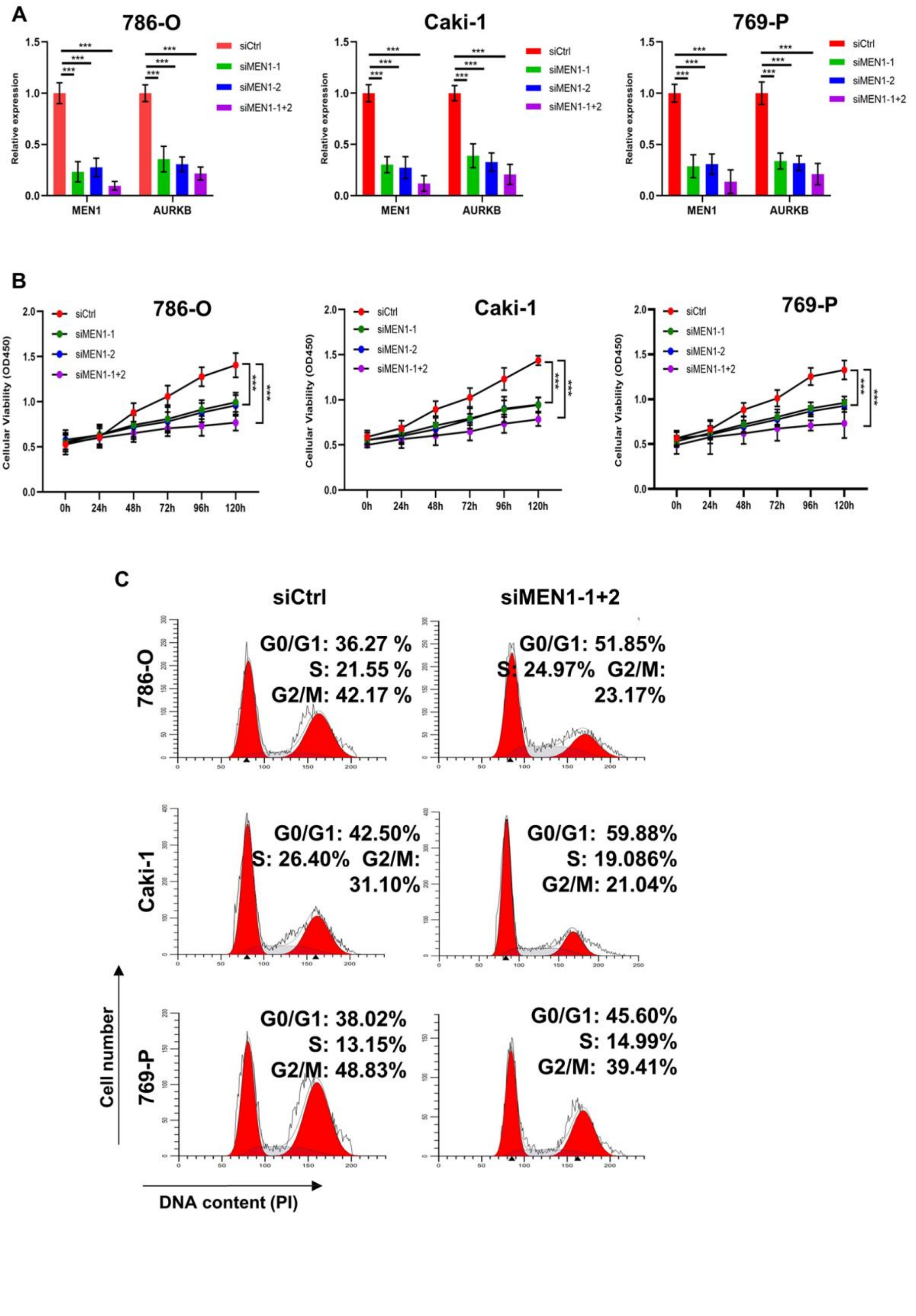

Supplementary Figure S8. Menin promotes the cell proliferation in ccRCC cells. **(A)** RT-qPCR analysis showing the AURKB expression upon the *MEN1* gene (MEN1-KD) by RNA interfering (siMEN1-1, siMEN1-2, siMEN1-1+2) or control (siCtrl) treatment in 786-O, Caki-1 and 769-P cells, respectively. **(B)** CCK-8 assay showing the effect of MEN1-KD (siMEN1-1, siMEN1-2, siMEN1-1+2) on the cell proliferation of the tested ccRCC cell lines. **(C)** MEN1-KD (siMEN1-1+2) induces cell cycle arrest at the G1 phase in ccRCC cells (786-O, Caki-1 or 769-P), compared to the cells transfected with siCtrl by flow cytometry. Percentages (%) of cell populations at different stages of cell cycles are listed within the panels. The experiment was repeated three times, and representative results are presented. The experiment was repeated three times, and representative results are presented. ns: no significant, * $P < 0.05$, ** $P < 0.01$, *** $P < 0.001$ vs control.

Supplementary Figure S9

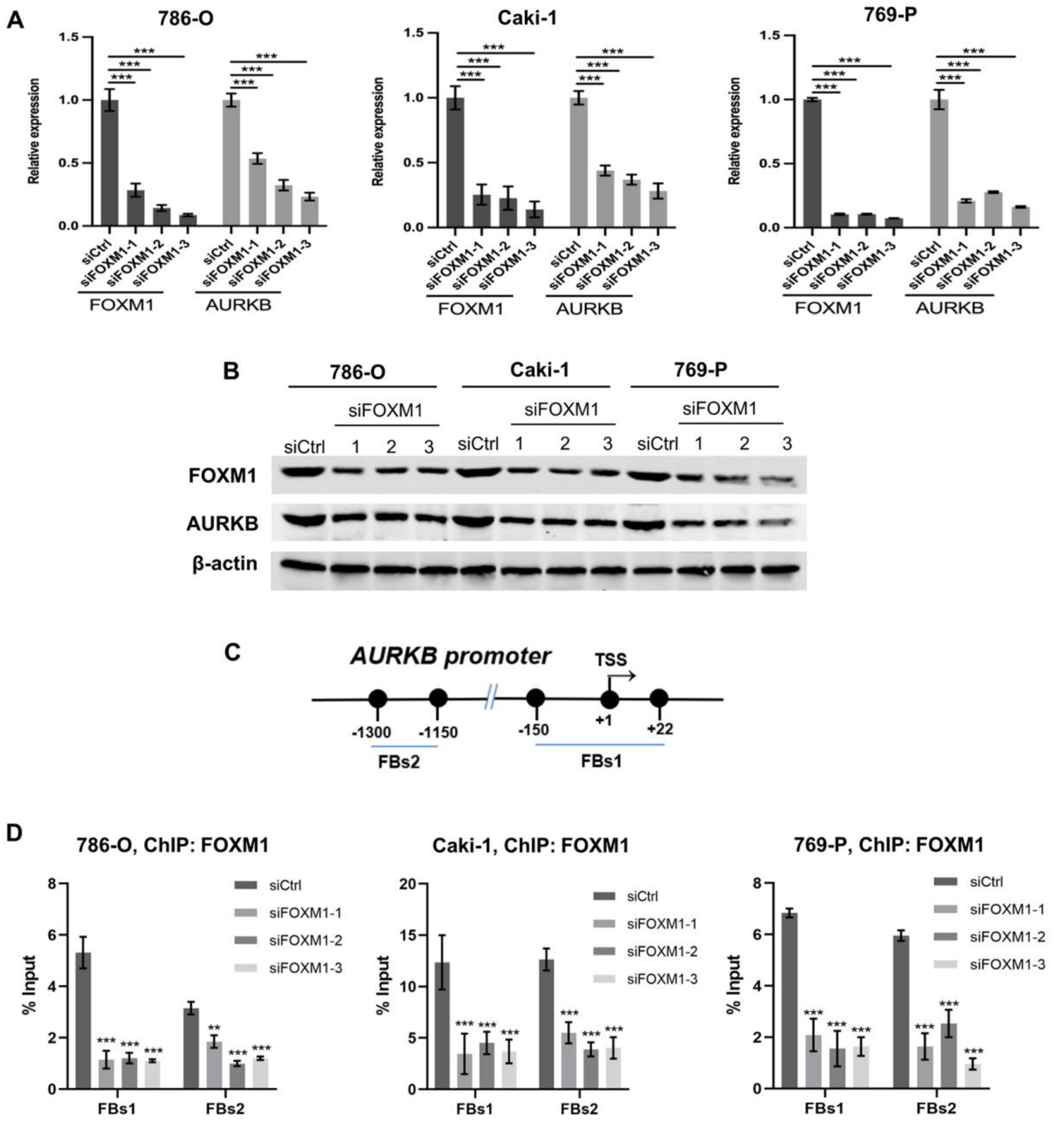

Supplementary Figure S9. FOXM1 is a potential target of TCF19 in ccRCC cells. **(A)** RT-qPCR assay showing the *FOXM1* and *AURKB* mRNA expression in TCF19-KD with siFOXM1-1, siFOXM1-2 and siFOXM1-3-treated ccRCC cell lines. **(B)** The FOXM1 and AURKB expression was analyzed by western blotting in FOXM1-KD 786-O, Caki-1 and 769-P cells. **(C)** Schematic representation of the FOXM1 binding site (FBs1 and FBs2) on the *AURKB* promoter. **(D)** ChIP-qPCR analysis evaluating the effect of FOXM1-KD on the level of FOXM1 recruitment to the *AURKB* promoter in siCtrl, siFOXM1-1, siFOXM1-2 and siFOXM1-3-treated 786-O, Caki-1 and 769-P cells. ns: no significant, * $P < 0.05$, ** $P < 0.01$, *** $P < 0.001$ vs control.

Supplementary Figure S10.

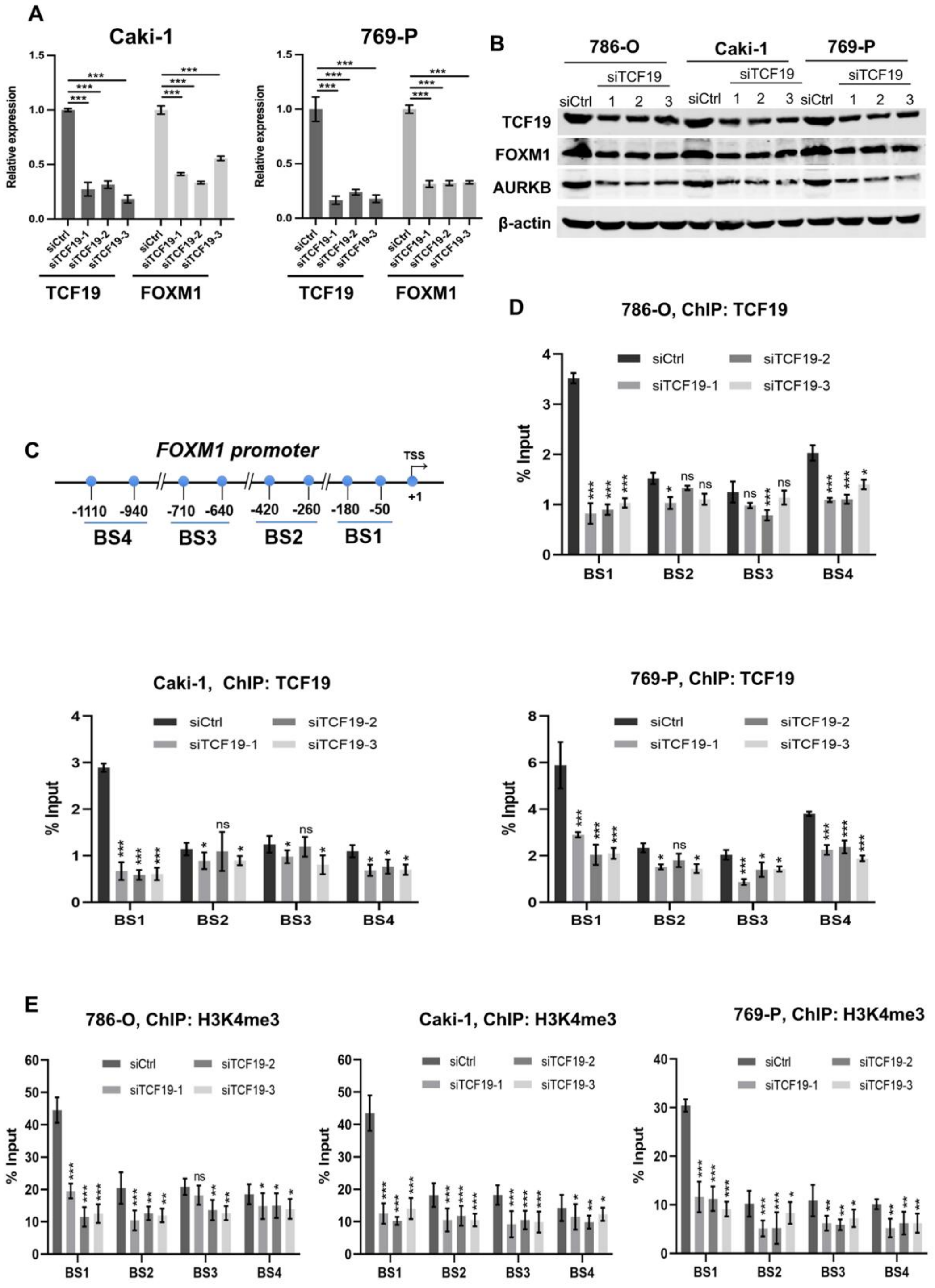

Supplementary Figure S10. FOXM1 regulates AURKB expression in ccRCC cells.

(A) RT-qPCR analysis showing the TCF19 and FOXM1 expression upon in siTCF19-1, siTCF19-2, siTCF19-3 or siCtrl-treated Caki-1 and 769-P cells, respectively. **(B)** Western blotting analyzed the expression of FOXM1 prot ein upon TCF19-KD or siCtrl treatment in ccRCC cell lines. **(C)** Schematic representation of the TCF19 binding site on the *FOXM1* promoter. **(D)** ChIP-qPCR was performed to assess the occupancy of TCF19 on the *FOXM1* promoter in siCtrl or siTCF19-1, siTCF19-2, siTCF19-3-treated Caki-1 and 769-P cells, respectively. **(E)** ChIP-qPCR analysis evaluating the effect of TCF19-KD on the level of H3K4me3 recruitment to the *FOXM1* promoter in siCtrl, siTCF19-1, siTCF19-2 and siTCF19-3-treated 786-O, Caki-1 and 769-P cells. ns: no significant, * $P < 0.05$, ** $P < 0.01$, *** $P < 0.001$ vs control.

Supplementary Figure S11.

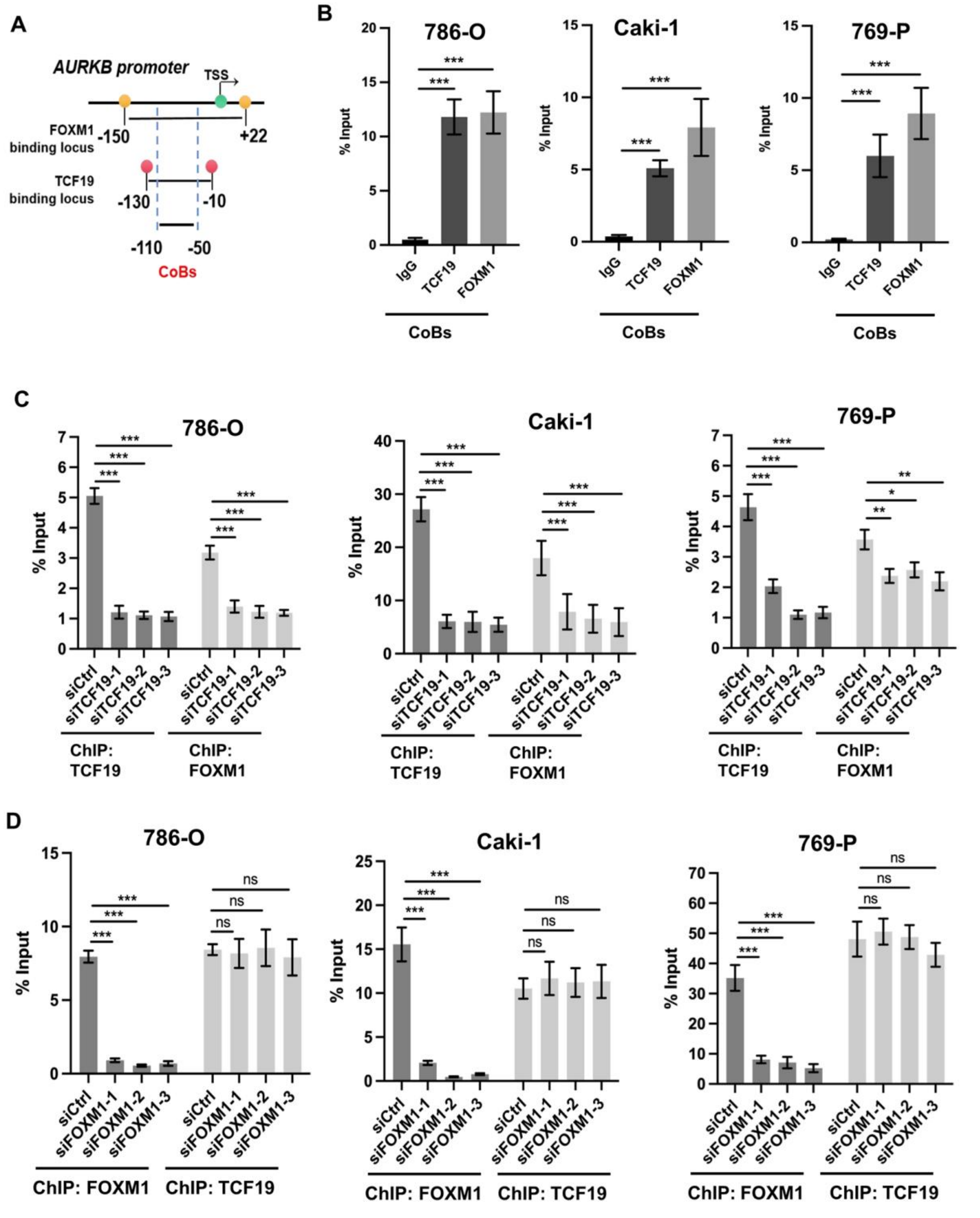

Supplementary Figure S11. TCF19 and FOXM1 co-regulate *AURKB* transcription in ccRCC cells.

(A) Schematic representation of the TCF19 and FOXM1 co-binding area (CoBs) on the *AURKB* promoter. **(B)** ChIP-qPCR assay to assess the occupancy of TCF19 and FOXM1 co-binding to the *AURKB* promoter in ccRCC cells. **(C)** ChIP-qPCR was performed to assess the influence of TCF19-KD on the TCF19 or FOXM1 occupancy on the TCF19 and FOXM1 co-binding area (CoBs) of *AURKB* promoter in siCtrl or siTCF19-1, siTCF19-2, siTCF19-3-treated 786-O, Caki-1 and 769-P cells. **(D)** ChIP-qPCR was performed to assess the influence of FOXM1-KD on the TCF19 or FOXM1 occupancy on the CoBs area of the AURKB promoter in siCtrl or siFOXM1-1, siFOXM1-2, siFOXM1-3-treated ccRCC cells, respectively. ns: no significant, * $P < 0.05$, ** $P < 0.01$, *** $P < 0.001$ vs control.

Supplementary Figure S12

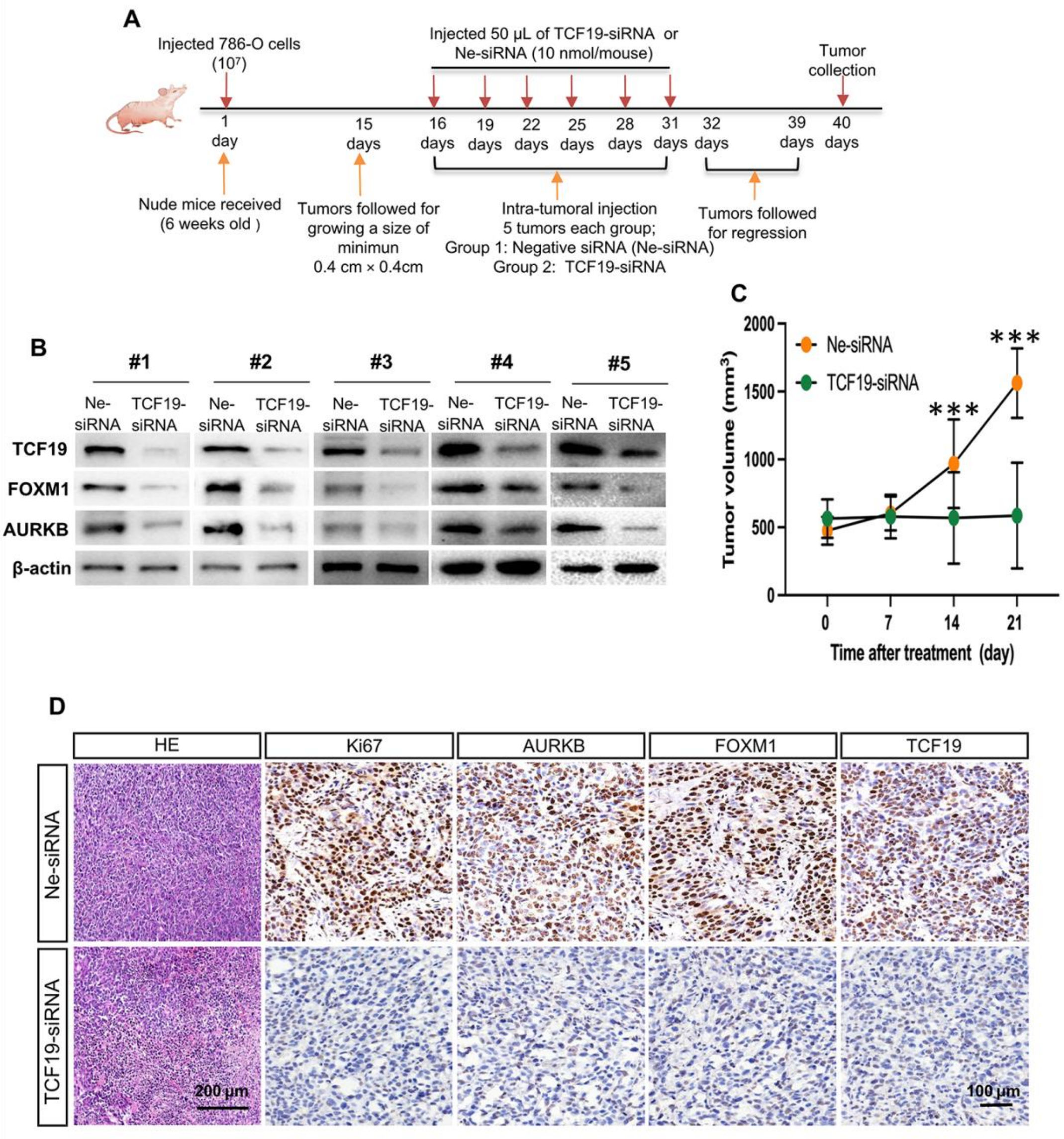

Supplementary Figure S12. The *TCF19* gene inactivation inhibits the proliferation of ccRCC cells in vivo.

(A) Schematic diagram of the strategy used for evaluating the effect of TCF19 on 786-O cell growth by xenografts in nude mice (n=5/group). **(B)** The TCF19, FOXM1 and AURKB expression in xenografts tumor tissues were detected by western blot assay in control group (Ne-siRNA) and the *TCF19* gene inactivation group (TCF19-siRNA). The Ne-siRNA and TCF19-siRNA from RiboBio (Guangzhou, China) were used for *in vivo* siRNA delivery. **(C)** Growth curve of xenograft tumor formed by indicated 786-O cells in nude mice. **(D)** Immunohistochemical (IHC) analysis showing Ki67, AURKB, FOXM1 and TCF19 expression in sh-control or sh-TCF19 xenografts tumor tissues.

Supplementary Figure S13

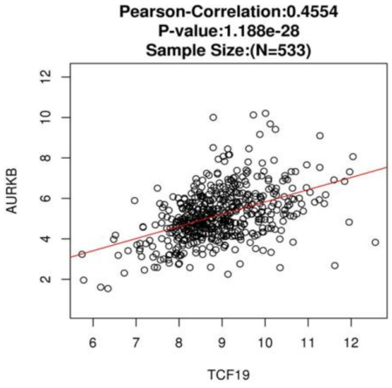

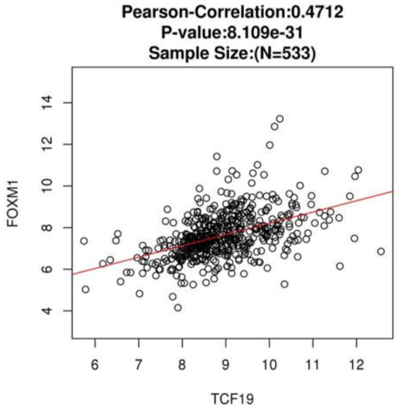

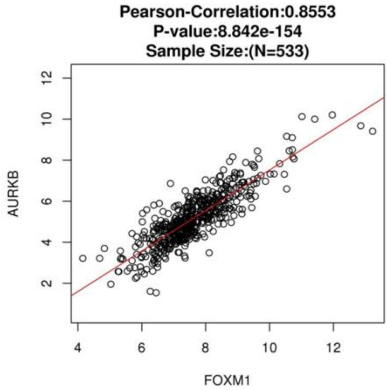

Supplementary Figure S13. Data mining analyses investigating the correlation between *TCF19* and *AURKB* mRNA expression, between *TCF19* and *FOXM1* mRNA expression and between *AURKB* and *FOXM1* mRNA expression using existing ccRCC database.
